# Structural Insights into the Mechanism of a Nanobody That Stabilizes PAI-1 and Modulates Its Activity

**DOI:** 10.3390/ijms21165859

**Published:** 2020-08-15

**Authors:** Machteld Sillen, Stephen D. Weeks, Sergei V. Strelkov, Paul J. Declerck

**Affiliations:** 1 Laboratory for Therapeutic and Diagnostic Antibodies, Department of Pharmaceutical and Pharmacological Sciences, KU Leuven, B-3000 Leuven, Belgium; machteld.sillen@kuleuven.be; 2 Laboratory for Biocrystallography, Department of Pharmaceutical and Pharmacological Sciences, KU Leuven, B-3000 Leuven, Belgium; sweeks@orthogontherapeutics.com (S.D.W); sergei.strelkov@kuleuven.be (S.V.S.)

**Keywords:** plasminogen activator inhibitor 1, cardiovascular disease, fibrinolysis, single-domain antibodies, nanobodies, protein–protein interaction, X-ray crystallography, small-angle X-ray scattering

## Abstract

Plasminogen activator inhibitor-1 (PAI-1) is the main physiological inhibitor of tissue-type (tPA) and urokinase-type (uPA) plasminogen activators (PAs). Apart from being critically involved in fibrinolysis and wound healing, emerging evidence indicates that PAI-1 plays an important role in many diseases, including cardiovascular disease, tissue fibrosis, and cancer. Targeting PAI-1 is therefore a promising therapeutic strategy in PAI-1 related pathologies. Despite ongoing efforts no PAI-1 inhibitors were approved to date for therapeutic use in humans. A better understanding of the molecular mechanisms of PAI-1 inhibition is therefore necessary to guide the rational design of PAI-1 modulators. Here, we present a 1.9 Å crystal structure of PAI-1 in complex with an inhibitory nanobody VHH-s-a93 (Nb93). Structural analysis in combination with biochemical characterization reveals that Nb93 directly interferes with PAI-1/PA complex formation and stabilizes the active conformation of the PAI-1 molecule.

## 1. Introduction

Plasminogen activator inhibitor-1 (PAI-1), a 45-kDa glycoprotein, is the main physiological inhibitor of plasminogen activators (PAs) such as tissue-type PA (tPA) and urokinase-type PA (uPA) [1]. As a member of the serine protease inhibitor (serpin) superfamily, the PAI-1/PA reaction follows the basic mechanism applied to all serpin/serine proteinase reactions [2]. Key to this reaction is that the PA recognizes PAI-1 as a (pseudo)substrate. To this end, PAI-1 carries a flexible surface-exposed reactive center loop (RCL) presenting a substrate-mimicking peptide sequence Arg346-Met347 (designated as P1-P1′). Initially PAs bind to PAI-1 through several exosite interactions to form a transient Michaelis complex [3,4]. Through subsequent cleavage of the P1-P1′ bond, an intermediate acyl-enzyme complex is formed. Following a branched pathway mechanism, the PAI-1/PA reaction can result either in the formation of an irreversible 1:1 stoichiometric complex or in the release of regenerated PA from cleaved PAI-1 [5]. In contrast to other serpins, PAI-1 has the unique ability to spontaneously convert into a stable latent form by inserting the RCL segment N-terminal to the P1-P1′ cleavage site (residues 331 to 346, designated as P16-P1) into the core of the protein. This transition occurs with a half-life of approximately two hours at 37 °C in vitro but is slightly longer in vivo due to the high-affinity association with vitronectin in plasma and the extracellular matrix [6,7].

Numerous studies have demonstrated that elevated levels of PAI-1 are a risk factor for various thrombotic diseases [8,9,10]. There is ample evidence that inhibition of PAI-1 activity results in a profibrinolytic effect [11,12,13,14,15]. Apart from its antifibrinolytic activity, PAI-1 can also influence tissue remodeling, and thus also plays a role in other pathologies such as fibrotic disorders and cancer [16,17]. The involvement of PAI-1 in various pathophysiological conditions makes it an attractive target for the development of specific inhibitors. The conformational plasticity of native PAI-1, which is critical for its function, however, makes it more difficult to identify and develop modulators of PAI-1 activity.

It has been shown that small molecules, peptides, monoclonal antibodies (mAbs), and antibody fragments such as nanobodies (Nbs) are able to modulate PAI-1 activity by interfering at different stages of the PAI-1/PA reaction. Antibodies can influence PAI-1 functionality in three possible ways: (I) by directly blocking the PAI-1/PA interaction [18,19,20], (II) by inducing the substrate behavior of PAI-1 [18,21], or (III) by accelerating the active to latent transition of the PAI-1 molecule [22]. mAbs and Nbs that possess the highest specificity and selectivity to PAI-1 are currently successfully used in translational studies [14,15]. Even though Nbs are ten times smaller than mAbs, they remain highly specific and selective and have several advantages over the use of mAbs, including higher stability and solubility, lower toxicity, excellent tissue penetration and easy manufacturing. Nbs can potentially serve as “building blocks” for multi-specific, multivalent or multi-paratopic molecules as they can easily be linked. Owing to these superior properties, Nbs are promising biotherapeutic candidates. Recently, we solved the crystal structures of the complexes formed by PAI-1 and two inhibitory Nbs, VHH-2g-42 (Nb42) and VHH-2w-64 (Nb64) [18]. These structures revealed in detail how both Nbs interact with the PAI-1 molecule and served as a starting point for targeted biochemical and biophysical research to confirm their mechanism of action. In this way, we revealed that Nb42 interferes with the initial PAI-1/PA complex formation, whereas Nb64 induces substrate behavior by redirecting the PAI-1/PA interaction to PAI-1 deactivation and regeneration of active PA.

Next to Nb42 and Nb64, VHH-s-a93 (Nb93) represents a different cluster within our nanobody library [23]. Nb93 was raised against and panned on a stabilized active PAI-1 variant (PAI-1-N150H-K154T-Q301P-Q319L-M354I, PAI-1-stab). In a plasminogen-coupled chromogenic assay, Nb93 showed to be a potent inhibitor of PAI-1 activity, reaching a maximum inhibition of glycosylated PAI-1-stab of approximately 76% with an IC_50_ value of 7 nM. Mutagenesis identified residues Glu242, Lys243, Glu244, and Glu350, located in the close vicinity of the RCL, as a part of the epitope. Furthermore, it was shown that Nb93 selectively binds the active conformation of PAI-1 with a dissociation constant (K_D_) in the low nanomolar range (0.3 to 6 nM) [23]. Notably, similar epitopes have been described for mAbs MA-42A2F6 (Lys243 and Glu350), MA-56A7C10 (Glu242, Lys243, Glu242, Glu350, Asp355, and Arg356) by mutagenesis [19,20]. The epitope of another neutralizing mAb MEDI-579 was revealed by X-ray crystallography [19]. The structure showed that the antigen-binding fragment (Fab) of MEDI-579 binds the RCL from P2 to P4′ (residues Ala345 to Glu350) and the adjacent exosites for binding to the so-called 37-loop (PAI-1 Tyr210, Glu212, and Glu350) and the 60-loop (PAI-1 Arg271 and Glu350) of PAs. Importantly, MA-42A2F6, MA-567C10, and MEDI-579 decrease or completely prevent PAI-1/PA complex formation. Since the epitopes of these mAbs and Nb93 may partially overlap, it was hypothesized that Nb93 might interfere with the complex formation between PAI-1 and PAs as well.

In this study, we report and discuss the crystal structure of the stabilized active PAI-1-W175F variant in complex with Nb93. This structure reveals a third and distinct mechanism by which a Nb can interfere with the PAI-1/PA reaction.

## 2. Results

### 2.1. Crystallographic Analysis of the PAI-1/Nb93 Complex

**Figure 1:**
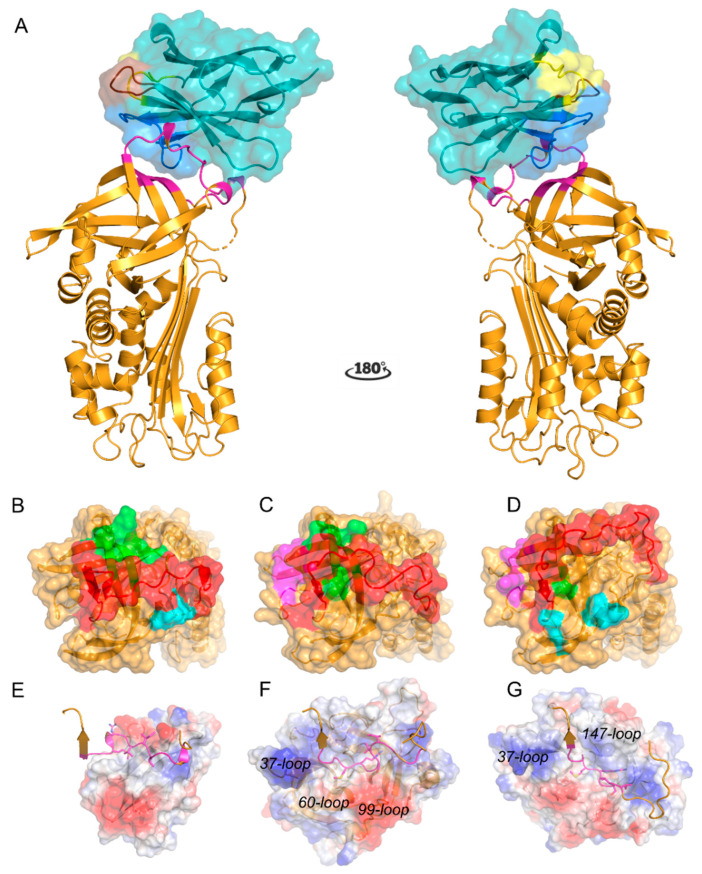
Crystal structure of the PAI-1/Nb93 complex. (**A**) Cartoon representation of the crystal structure of PAI-1 (orange) complexed with Nb93 (teal). Residues 331 and 332 of the reactive center loop (RCL) were not observed in the crystal structure and are presented by a dashed line. Together with the cartoon representation, the biological surface of Nb93 is represented. Framework regions of Nb93 are colored cyan and teal; the complementarity-determining regions (CDR) 1, CDR2, and CDR3 are in yellow, brown, and blue, respectively. The residues in PAI-1 closer than 4 Å to Nb93 are in magenta; (**B**) Surface representation of the top of the PAI-1 molecule with the indicated contact regions (residues closer than 4 Å) for Nb93. Nb93 binds to the RCL in red and has adjacent contact regions in green (Thr205, Lys207, and Pro270) and cyan (Asp181 and Ser182); (**C**), Surface representation of the top of the PAI-1 molecule with the indicated contact regions (residues closer than 4 Å) for tissue-type plasminogen activator (tPA). The RCL is in red, the exosite region for the 37-loop of tPA is in magenta (Tyr210, Glu212, Asp222, andTyr241) and the contact regions adjacent to the RCL are in green (Thr205, Lys207, and 269-Leu-Pro-Arg-Leu-272); (**D**) Surface representation of the top of the PAI-1 molecule with the indicated contact regions (residues closer than 4 Å) for urokinase-type plasminogen activator (uPA). The RCL is in red, the exosite region for the 37-loop of uPA is in magenta and the contact regions adjacent to the RCL are in green (Leu272) and cyan (Ser182, Ser183, and Arg187); (**E–G**) Cartoon representation of the bound RCL and the charged surface representation of Nb93 (**E**), tPA (**F**), and uPA (**G**), respectively. Exosite loops of tPA and uPA are indicated.

The crystal structure of the binary complex PAI-1-W175F/N93 (PDB ID 6ZRV) was determined to 1.88 Å resolution with an *R_work_* of 19.7% and an *R_free_* of 23.3% (Table 1). The asymmetric unit contains a single PAI-1/Nb93 complex. The structure of PAI-1 in the complex (Figure 1A) shows the evolutionarily conserved serpin topology consisting of three β-sheets (A–C) and nine α-helices (hA–hI) [2]. The first six N-terminal residues (VHHPPS) as well as residues Ser331 and Gly332 of the RCL were disordered in the electron density maps and are therefore not included in the final model. Comparison of the PAI-1-W175F structure in the complex and the isolated PAI-1-W175F structure (PDB ID 3Q02, chain A) shows that the conformation of PAI-1 is unaltered by interacting with Nb93 (Cα RMSD of 1.197 Å, excluding the flexible RCL residues 331–356). Nb93 adopts the typical immunoglobulin fold, consisting of four framework regions (FR1–FR4) that form the β-sandwich core structure of the immunoglobulin domain. This domain is held together by a disulfide bond connecting Cys22 and Cys96 in FR1 and FR3, respectively. As often observed in Nbs, Nb93 features a 15-residue-long CDR3 which is stabilized by an additional interloop disulfide bond formed between a non-canonical Cys at position 50 located in FR2 and Cys120 located approximately in the middle of the CDR3 loop [18,24].

### 2.2. Nb93 Binds to the Surface-Exposed RCL and Thereby Directly Competes for the PA Binding Site on PAI-1

**Figure 2:**
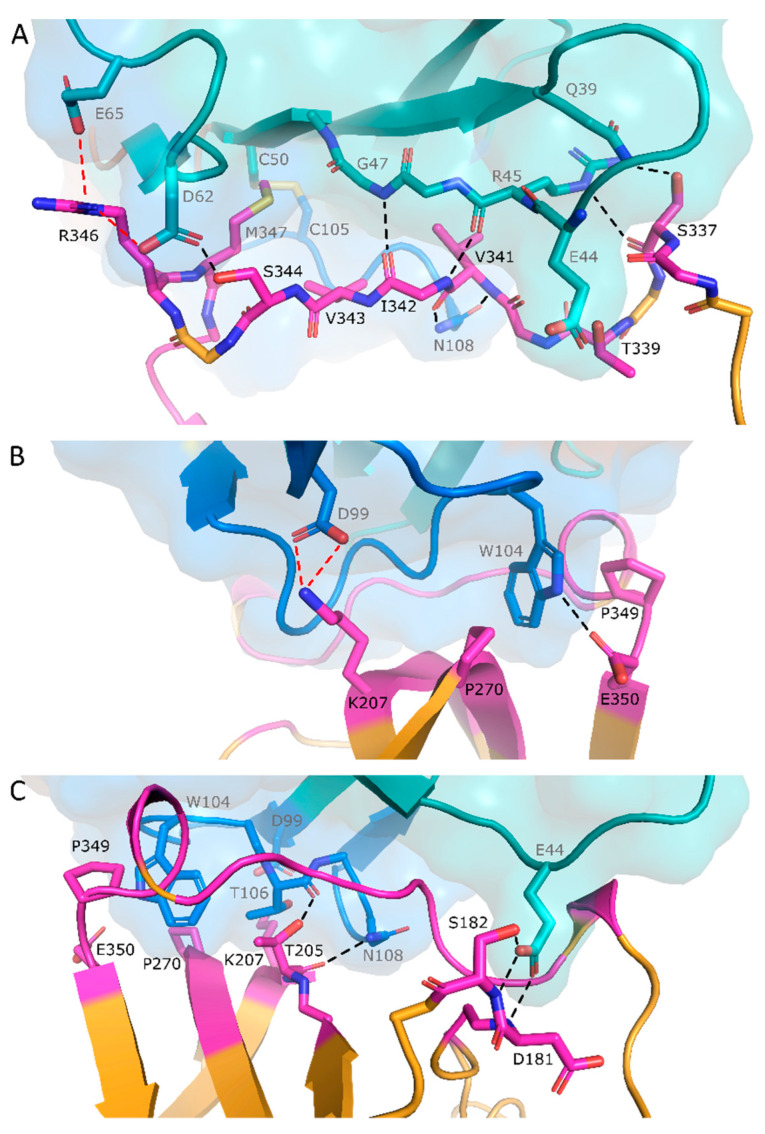
Interaction interface between PAI-1 and Nb93. (**A**) Detail of the interaction between Nb93 and the RCL of PAI-1; (**B**,**C**) Detail of the interaction between Nb93 and residues located in the direct environment of the RCL of PAI-1. PAI-1 is colored orange, with residues closer than 4 Å to Nb93 in magenta. Framework regions of Nb93 are in teal; CDR1, CDR2, and CDR3 are in yellow, brown, and blue, respectively. Hydrogen bonds are indicated by black dotted lines; salt bridges are indicated by red dotted lines.

The structure reveals that Nb93 binds a large part of the surface-exposed RCL (residues 337 to 350), including the bait peptide bond P1-P1′ (Figure 1A and Figure 2A). Nb93 has its CDR3 loop folded over the FR2 region and binds PAI-1 in a sideways orientation, thereby enfolding the RCL in the groove formed between CDR3 and FR2 and FR3 (Figure 1A). The parallel orientation of the RCL to the 5-stranded β-sheet of Nb93 results in an interface of 948 Å^2^. The interaction is stabilized by several backbone and side chain hydrogen bonds as well as charge-mediated interactions that cumulatively contribute to the specific high affinity binding (Figure 2 and Table 2). In this respect, backbone hydrogen bonds are formed between Nb93 FR2 Arg45 and Gly47 and PAI-1 RCL Ile342 (Figure 2A). Hydrogen bonds involving side chains are established between Nb93 FR2 Gln39 and PAI-1 RCL Ser337, as well as between Nb93 FR3 Asp62 and PAI-1 RCL Ser334 (Figure 2A). Furthermore, through the side chains of Trp104 and Asn108 in CDR3, Nb93 forms hydrogen bonds with the side chain of Glu350 and the backbone of Val341 in PAI-1 (Figure 2A,B). Notably, Trp104 in the Nb93 CDR3 loop interacts with a small pocket where it is sandwiched between Pro270 in strand 2 of β-sheet C (s2C) and Pro349 in the RCL of PAI-1. This way, Trp104 is in an optimal position to participate in hydrogen bonding with the side chain of Glu350 (Figure 2B). Importantly, the binding region on the PAI-1 RCL also includes the P1-P1′ cleavage site. Indeed, Asp62 and Glu65 in FR3 of Nb93 interact with Arg346 (P1) through the formation of two salt bridges (Figure 2A). Furthermore, P1′ residue Met347 fits into a small hydrophobic cavity formed between the side chains of Nb93 residues Tyr59, Trp104, Phe107, and the disulfide bond formed between Cys50–Cys105 (Figure 2A). Additionally, through interactions with residues in the direct environment of the RCL, Nb93 tightly anchors the flexible loop to the top side of the PAI-1 molecule (Figure 1B). These interactions include hydrogen bonds formed between Nb93 FR2 Glu44 and PAI-1 Asp181 and Ser182 as well as between Nb93 CDR3 Thr106 and Asn108 and PAI-1 Thr205, and a salt bridge between Nb93 FR3 Asp99 and PAI-1 Lys207 (Figure 2A,B).

Of note, the crystal structure of the PAI-1/Nb complex provides a rational explanation for the ability of Nb93 to inhibit PAI-1/PA interactions. The previously established structures of the PAI-1/tPA and PAI-1/uPA Michaelis complexes revealed that residues 338 to 352 of the RCL are inserted into the catalytic site cleft of tPA and uPA (Figure 1C,D) [3,4]. Furthermore, a hydrophobic cluster within the RCL (Val341-Ile342-Val343) binds to the hydrophobic S1β pocket of tPA, which is blocked by Gln192 in unbound tPA. Upon binding to PAI-1, Gln192 of tPA interacts with PAI-1 Thr205 (located on strand 3 of β-sheet C) and PAI-1 Pro349 (located in the RCL) and therefore acts as a switch to open up the S1β pocket. Apart from the interactions with the RCL, interactions with PAs are also mediated through exosites on the PAI-1 molecule. Whereas the positively charged 37-loop of PAs is important for making extensive interactions with a negatively charged patch located close to the C-terminal part of the RCL on PAI-1, the tPA 60-loop and 99-loop form a negatively charged region on tPA to make contact with Lys207 in PAI-1 (Figure 1C) [3]. In contrast to tPA, the uPA 147-loop interacts with residues Ser183 and Arg187 outside the RCL (Figure 1D) [4]. In this respect, it is notable that the PAI-1/Nb93 interface includes interactions with the above mentioned hydrophobic cluster, Asp181, Ser182, Thr205, Lys207, and Pro349 (Figure 1B). As a result, Nb93 binds PAI-1 in a protease-like manner by directly interacting with the PAI-1 RCL and by combining the exosite binding mechanisms of both PAs, i.e., by interacting with residues that are normally engaged in exosite interactions with the 60- and 99-loop of tPA and the 147-loop of uPA.

**Figure 3:**
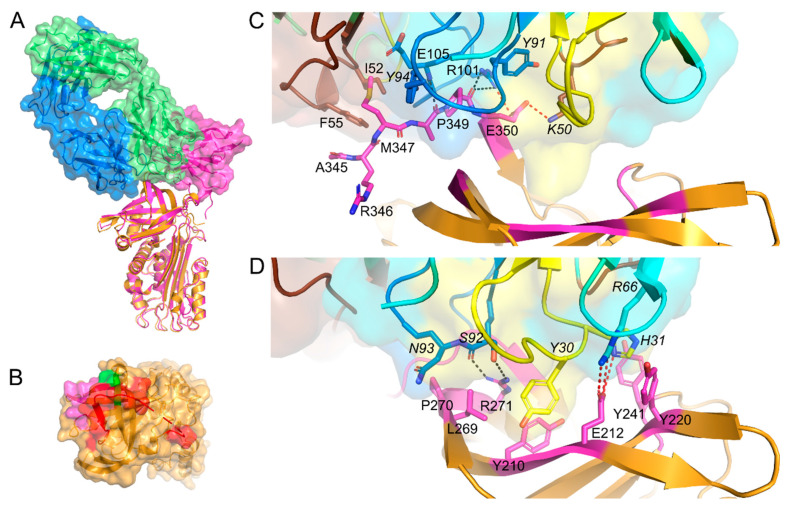
Crystal structures of the PAI-1/Nb93 complex and the PAI-1/Fab-MEDI-579 complex. (**A**) Superimposition of the structures of the PAI-1/Nb93 (magenta) complex and PAI-1/Fab-MEDI-579 complex (PDB ID 6I8S) [19]. In the latter complex PAI-1 is orange; the heavy and the light chain of the Fab fragment are colored green and blue, respectively. Together with the cartoon representation, the biological surfaces of Nb93 and the Fab fragment are represented; (**B**) Surface representation of the top of the PAI-1 molecule with the indicated contact regions (residues closer than 4 Å) for MEDI-579. MEDI-579 binds to the C-terminal part of the RCL in red and has adjacent contact regions in magenta (Tyr210, Glu212, Tyr220, and Tyr241) and green (Leu269, Pro270, and Arg271); (**C**) Detail of the interaction between MEDI-579 and the RCL of PAI-1; (**D**) Detail of the interaction between MEDI-579 and residues located in the direct environment of the RCL of PAI-1. PAI-1 is colored orange, with residues closer than 4 Å to MEDI-579 in magenta. Framework regions of MEDI-579 are in green and cyan for the heavy and light chain of the Fab fragment, respectively; CDR1, CDR2, and CDR3 of the variable heavy (V_H_) and variable light (V_L_) domain are colored yellow, brown, and blue, respectively. Residues located in the V_L_ domain are cursive. Hydrogen bonds and salt bridges are indicated by black and red dotted lines, respectively.

Superimposition of the current structure and the published structure of PAI-1 in complex with the Fab fragment of MEDI-579 (PDB ID 6I8S) [19] shows that their respective PAI-1 epitopes only partially overlap (Figure 3A). Whereas Nb93 tightly enfolds the RCL in the groove formed between CDR3 and the β-barrel core, MEDI-579 binds the RCL across the groove between the variable heavy and the variable light domain. Both Nb93 and MEDI-579 anchor the RCL to the body of the PAI-1 molecule via interactions with previously described exosites for PAs (Figure 3B). However, whereas the contact region for MEDI-579 is concentrated around the C-terminal region of the RCL (P2 to P4′, Figure 3C) and neighboring exosites for the 37-loop and 60-loop of PAs (Figure 3D), interactions between PAI-1 and Nb93 involve almost the entire PAI-1 RCL (P10-P4′, Figure 2A) and exosites adjacent to both the N- (60- and 99-loop) and C-terminal (147-loop) part of the RCL (Figure 2B,C).

### 2.3. The Structure of the PAI-1/Nb93 Complex Supports Previously Obtained Data on the Affinity Profile of Nb93

The crystal structure of the PAI-1/Nb93 complex rationally explains the observed specificity of Nb93, including the absence of cross-reactivity with mouse and rat PAI-1. The crucial involvement of the RCL is also in agreement with the observed selectivity of Nb93 for the active conformation of PAI-1. Previous affinity determination using surface plasmon resonance showed a lack of binding to latent PAI-1 and two latent variants of PAI-1, PAI-1-H185A-R186A-H187A, or PAI-1-D89A-T94A [25]. The current structure reveals that these residues do not reside within the epitope of Nb93, with the closest residue His185 located approximately 17 Å away from the proposed epitope of Nb93. Therefore, the latency behavior of both variants most likely lies at the basis of the observed affinity loss towards these mutants. Furthermore, a reduced affinity towards PAI-1-EKE242-4AAA-E350A was observed. As mentioned, residue Glu350 is involved in hydrogen bonding with Trp104 in CDR3 of Nb93. Residues 242–244 are located in between the exosite for the 37-loop of PAs and the gate region through which the C-terminal part of the RCL has to move during loop insertion [26]. However, based on the structure, residues 242–244 do not directly engage in the interaction with Nb93. The affinity determination also showed no binding to mouse and rat PAI-1. Notably, Glu350 is not conserved in rodent PAI-1. Furthermore, the hydrophobic cluster Val341-Ile342-Val343, which interacts with the groove between Nb93 CDR3 and FR2 and FR3, has a different composition of hydrophobic residues in mouse (Phe341-Val342-Ile343) and rat (Ile341-Leu342-Val343) PAI-1. Other non-conserved residues belonging to the epitope include (human positions first, mouse/rat last) Asp181Glu/Glu, Ser182Ala/Ala, and Thr205Ser/Asn.

### 2.4. The X-Ray Structure Is Valid in Solution

**Figure 4:**
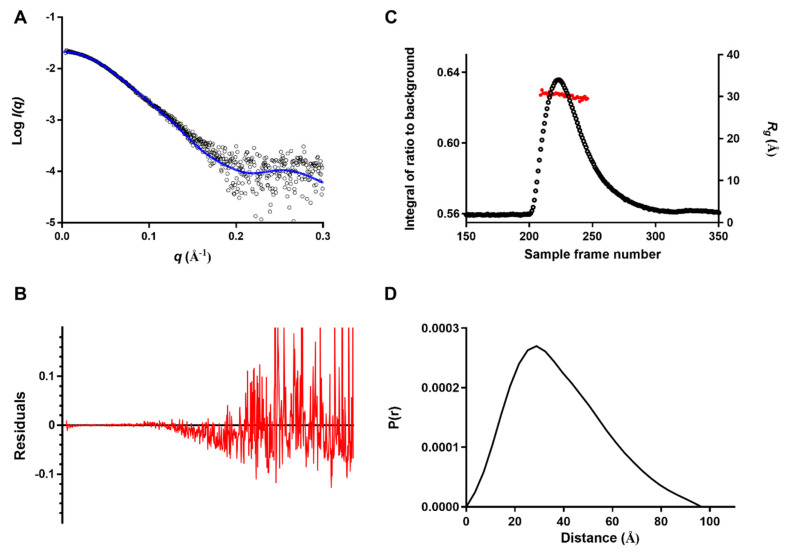
Small-angle X-ray scattering (SAXS) data for the PAI-1/Nb93 complex. (**A**) The averaged scattering curve (black circles) was fitted with the theoretical scattering curve calculated using CRYSOL from the crystal structure of the complex (blue line); (**B**) The residual plot corresponding to the fit is represented by the red curve. (**C**) In total, 480 frames were collected during elution from an Agilent Bio SEC-3, 300 Å, 4.6 × 300 mm column. ScÅtter was used to plot the *R_g_* as a red circle scaled on the right axis, the integral of ratio to background is shown as a black circle and scaled on the left axis. (**D**) The pair distribution function P(r) was solved using ScÅtter incorporating the averaged scattering curve data up to *q* = 0.29 Å^−1^.

To verify whether the crystallographic PAI-1/Nb93 complex is also true in solution, small-angle X-ray scattering (SAXS) was carried out. The theoretical scattering profile of the crystallographic PAI-1/Nb93 complex provides a good fit to the experimental SAXS curve, with a goodness of fit χ2 of 1.4 (Figure 4A,B). The complex showed a stable *R_g_* of approximately 30 Å across the elution peak (Figure 4C), underscoring the homogeneity of the sample. The complex in the sample had a calculated mass of 57.8 kDa based on the Porod volume of 95,870 Å^3^, which is in excellent agreement with the expected mass of 56 kDa. Furthermore, the curve of the pair distribution function showed a *D_max_* of 96.5 Å (Figure 4D), which corresponds to the maximum dimension of 94 Å calculated based on the crystal structure of the complex.

### 2.5. Nb93 Exhibits a Biphasic Effect on the Inhibition of PAI-1 Activity Due to a Substantial Stabilization of the Active PAI-1 Conformation

**Figure 5:**
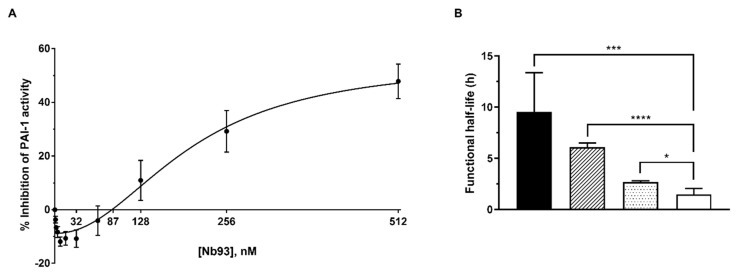
Evaluation of the inhibitory and stabilizing effects of Nb93 on active PAI-1. (**A**) Biphasic dose–dependent response of Nb93 on wild-type PAI-1 (PAI-1-wt) activity, characterized by a stabilizing effect at concentrations below and an inhibitory effect at concentrations above 87 nM; (**B**) The functional half-life of active PAI-1-wt in the absence (open bars) or the presence of 64 nM Nb93 (black bars), 32 nM Nb93 (hatched bars) or 64 nM Nb42 (dotted bars). All data are represented as mean ± SD; *n* ≥ 3. * *p* < 0.05, *** *p* < 0.001, **** *p* < 0.0001.

Interestingly, Nb93 exhibits a biphasic dose–response phenomenon characterized by a stabilizing effect at low Nb doses and an inhibitory effect at high doses (Figure 5A). At concentrations below 87 nM, Nb93 was able to preserve up to 10% of PAI-1 activity in the current experimental setting. This stabilization was further investigated by analyzing the effect of Nb93 on the rate of auto-inactivation of active PAI-1. Therefore, PAI-1-wt was incubated at 37 °C with either PBS buffer or Nb93 at two different concentrations (32 or 64 nM). As a control, 64 nM Nb42, which binds to an exosite for PAs located in strand 1 and 2 of β-sheet B (s1B-s2B) remote from the RCL, was also incubated with PAI-1-wt. As expected, in the absence of Nbs, PAI-1-wt showed a functional half-life of 1.5 ± 0.6 h. Binding of Nb42 only caused a small increase in the functional half-life (3 ± 0.15 h, *p* = 0.0102, Figure 5B). Nb93, which directly binds to the RCL, remarkably stabilized the active conformation of PAI-1 at both 32 nM and 64 nM concentration, resulting in prolonged half-lives of 6 ± 0.4 h (*p* = 0.000004) and 10 ± 4 h (*p* = 0.0003), respectively, as compared to PAI-1-wt. In contrast, at Nb93 concentrations exceeding 87 nM, inhibition of PAI-1 activity becomes increasingly important, reaching a maximum inhibition of 55 ± 9% with an IC_50_ value of 202 ± 41 nM (Figure 5A).

## 3. Discussion

Apart from its role in normal physiological processes, including fibrinolysis and wound healing, PAI-1 has also been associated with many acute and chronic pathological processes such as cardiovascular disease, tissue fibrosis, cancer and type 2 diabetes [27,28,29,30]. Therefore, a significant clinical interest is tied to PAI-1 as a putative drug target for the treatment of PAI-1-related pathophysiological conditions. However, despite the many efforts to develop PAI-1 inhibitors, there are no PAI-1 inhibitors that to date have been clinically approved, presumably because of toxicity and selectivity issues [31]. A better understanding of the molecular mechanisms of PAI-1 inhibition is therefore necessary to guide the rational design of PAI-1 modulators.

Here, we report the structural characterization of the interaction between PAI-1 and a neutralizing nanobody, Nb93. The epitope observed in the structure of the PAI-1/Nb93 complex comprises a major part of the RCL (residues 337 to 350) as well as some residues on β-sheet C (Asp181, Ser182, Thr205, and Lys207) located in close proximity to the RCL. Zhou et al. previously determined the epitope of Nb93 by alanine scanning and identified residues Glu242, Lys243, Glu244, and Glu350, located near the RCL and the exosite for the 37-loop of PAs, as a part of the epitope [23]. Indeed, we were able to confirm the involvement of Glu350 by structural analysis of the PAI-1/Nb93 complex. Residues 242 to 244, however, are located more than 4 Å away from Nb93 in the complex. Since our structure does not indicate a direct contribution of these residues to Nb93 binding, this suggests that the Glu350 mutation contributes more towards the observed overall loss in affinity. Furthermore, due to their localization close to the RCL, mutations in this region may affect the conformation of the flexible RCL and thus be important for the integrity of the structural epitope. It is also important to keep in mind that, considering the difference between the functional epitope as determined by alanine-scanning mutagenesis (i.e., mainly the distal C-terminal part of the RCL) and the structural epitope (i.e., both the proximal N-terminal and distal C-terminal part of the RCL), one must be careful in interpreting mutagenesis results. This study underscores the importance of structural analysis and additional biochemical characterization of the interactions between PAI-1 and its inhibitors. In this respect, it is possible that mAbs MA-42A2F6 and MA56A7C10 bind PAI-1 through a comparable mechanism as for Nb93, since mutagenesis indicated a similar epitope in PAI-1 [20].

Importantly, binding of Nb93 to the PAI-1 RCL, including P1-P1′, while anchoring it to the top of the molecule is in agreement with the ability of Nb93 to prevent the PAI-1/PA interaction as well as its ability to stabilize the active conformation of the PAI-1 molecule. Even though there is a difference in the conformation of the RCL in the PAI-1/Nb93 complex as compared to the PAI-1/PA complexes [3,4], our structure shows that Nb93 binds PAI-1 in a protease-like manner. Instead of the RCL being inserted into the catalytic site cleft of the PA, the RCL binds into the groove formed between CDR3 and FR2 and FR3 and takes on a parallel orientation to the β-barrel core of Nb93. By interacting with residues adjacent to the RCL, which normally take part in exosite interactions with tPA and uPA, Nb93 anchors the RCL to the top of the PAI-1 molecule. Several residues in the PAI-1/Nb93 interaction interface, including RCL residues 337 to 350, Thr205, and Lys207, are also identified as residues directly engaged in the binding to PAs [3,4]. Furthermore, Nb93 makes contact with residues Asp181 and Ser182 neighboring the contact region (Ser183 and Arg187) for the 147-loop of uPA. Therefore, our structure supports a directly competitive mechanism of action for Nb93, i.e., by directly blocking the binding sites for PAs. A similar mechanism was revealed for mAb MEDI-579 by structural analysis of the complex between its Fab fragment and PAI-1 [19]. Here, a major part of the RCL binds in the cleft between the variable heavy and variable light domain of the Fab fragment. However, the binding region for Nb93 and MEDI-579 in PAI-1 only partially overlap. Nb93 combines the exosite binding mechanisms of both PAs, i.e., by having similar contact regions as the 60- and 99-loop of tPA and the 147-loop of uPA, whereas MEDI-579 interacts with the binding region for the 37-loop and the 60-loop of PAs [19].

The current structure also provides a rational explanation for the observed specificity of Nb93 for the active conformation of PAI-1 and its ability to stabilize this conformation [23]. Previously, a catalytically inactive mutant of tPA, tPA-Ser195Ala, was shown to stabilize the active conformation of PAI-1 in cultured human umbilical vein endothelial cells due to the formation of a noncovalent complex following secretion of PAI-1 [32]. Similarly, binding of Nb93 disfavors RCL insertion, which is required for spontaneous latency transition and leads to a 10-fold increase in the functional half-life of active PAI-1. As a consequence, Nb93 shows a biphasic dose–response effect characterized by stabilization at low Nb93 concentrations and an increased inhibitory effect associated with rising Nb93 concentrations. Together with the lack of rodent cross-reactivity, this may limit the development and in vivo use of Nb93 as a therapeutic. However, due to its high affinity and preferential binding to the active form of PAI-1, it may be further developed rather as a nanobody-based diagnostic or analytical tool, e.g., to selectively purify and stabilize the active form PAI-1 or to quantify active PAI-1 in an ELISA setting. Interestingly, a similar binding mechanism, i.e., in which a short linear peptide is bound into the groove between the CDR3 and the framework regions of the Nb, has been reported for a few other Nbs that have been further developed for biotechnical and bioanalytical applications [33,34]. In this respect, it could be of interest to investigate whether Nb93 can bind solely to the surface-exposed portion of the RCL of PAI-1 (residues 333–350). This may open up new perspectives to further develop Nb93 as an affinity capture reagent [35], as a crystallization chaperone for proteins supplied with a peptide tag [36] or as a diagnostic tool.

## 4. Materials and Methods

### 4.1. Cloning, Expression, and Purification of PAI-1 Variants and Nb93

A Nb library was previously constructed, and a panel of Nbs with distinct inhibitory properties towards PAI-1 activity was characterized and reported [23]. Sequences encoding Nb93, non-glycosylated wild-type PAI-1 (PAI-1-wt), and PAI-1-W175F were cloned into the cytoplasmic expression vector pETHSUK2, which includes a cleavable N-terminal His_6_SUMO-tag [37].

Overexpression of all constructs was carried out in *Escherichia coli* Rosetta 2(DE3)pLysS strain (Merck, Darmstadt, Germany). Transformed colonies were initially cultured at 37 °C in the Luria-Bertani medium for 8 h. Transformed colonies were initially cultured in Luria-Bertani medium for 8 h at 37 °C. A 500 µL aliquot of the preculture was subsequently used to inoculate 1 L of ZYP-5052 auto-induction medium supplemented with 1 mL SE15 antifoam (Sigma-Aldrich, Darmstadt, Germany), after which the culture was incubated overnight at 24 °C. Once the culture reached an OD_600_ of 4, the temperature was reduced to 18 °C to allow the cultures to grow for another 24 h. Cells were harvested by centrifugation at 8000 RPM and cell pellets were resuspended in IMAC 12.5 buffer (50 mM sodium phosphate, 250 mM sodium chloride, 12.5 mM imidazole, pH 7 (PAI-1 variants) or pH 7.5 (Nb93)) to which 10 mM magnesium chloride and 100 units Cryonase (Takara Bio Europe, Saint-Germain-en-Laye, France) were added. The suspension was sonicated 3 times by 1 s on/1 s off pulses at 70% amplitude, with a 1 min total pulse time using the Branson 450 digital sonifier (Branson Ultrasonics, Danbury, Connecticut, United States), with a 30 min rest on ice between cycles. Insoluble components, including cell debris and inclusion bodies, were removed by centrifugation at 13,000 RPM at 4 °C for 45 min. The resulting supernatant containing the His_6_SUMO-tagged PAI-1 variants was loaded on a 5 mL HisTrap HP column (GE Healthcare, Chicago, Illinois, United States). Following loading, the column was washed with 5 column volumes (CV) of IMAC 12.5 buffer before eluting the protein using an IMAC buffer containing 500 mM imidazole. The fractions containing the SUMO-fusion protein were combined and an excess of SUMO hydrolase (SH) was added at a 1:250 mass ratio. The mixture was dialyzed overnight at 4 °C with two changes against 1 L of dialysis buffer (20 mM sodium phosphate buffer pH 6, 250 mM NaCl) and subsequently loaded on a 10 mL HiTrap SP column (GE Healthcare) pre-equilibrated with IEX A buffer (20 mM sodium phosphate pH 6, 250 mM NaCl). Following a 5 CV wash step with IEX buffer A, the PAI-1 variant was eluted using a gradient from 0 to 40% with IEX buffer B (20 mM sodium phosphate pH 6, 1 M NaCl). Protein-containing fractions were combined and concentrated using a Vivaspin 15R with a 10 kDa molecular-mass cutoff (Sartorius, Göttingen, Germany) and further purified by gel filtration on a HiLoad^®^ 16/600 Superdex^®^ 200 pg column (GE Healthcare) equilibrated in a BIS-TRIS buffer for crystallization (20 mM BIS-TRIS pH 6, 300 mM NaCl).

His_6_SUMO-tagged Nb93 was recovered from the pellet by solubilizing the washed inclusion bodies in IMAC 12.5 pH 7.5 buffer containing 5 M urea. To refold Nb93, the solubilized protein was dialyzed overnight at 4 °C with two changes against of 1 L of dialysis buffer (IMAC 12.5 pH 7.5). The refolded fusion protein was purified using a 5 mL HisTrap HP column (GE Healthcare) and treated with SUMO-hydrolase to remove the His_6_-tagged SUMO domain similarly as the PAI-1 variants. Subsequently, this sample was dialyzed overnight at 4 °C with two changes of 1 L IMAC 12.5 pH 7.5 and reapplied on a 5 mL HisTrap HP column (GE Healthcare). The flowthrough containing non-tagged Nb93 was collected and concentrated using a Vivaspin 15R with a 5 kDa molecular-mass cutoff (Sartorius). Finally, Nb93 was further purified by gel filtration on a HiLoad^®^ 16/600 Superdex^®^ 200 pg column (GE Healthcare) equilibrated either in a BIS-TRIS buffer for crystallization with PAI-1 (20 mM BIS-TRIS pH 6, 300 mM NaCl) or PBS buffer at pH 7.4 for biochemical characterization. All purification steps were conducted on ice or at 4 °C.

### 4.2. Crystallization and Data Collection

To prepare the PAI-1-W175F/Nb93 complex, PAI-1-W175F was mixed with Nb93 at a 1:1.1 molar ratio and incubated at 22 °C for 30 min. The solution of the complex was concentrated to 10.4 mg/mL using a Vivaspin with a 5 kDa molecular-mass cutoff (Sartorius) at 4 °C. The PAI-1-W175F/Nb93 complex was crystallized in sitting drops by mixing 200 nL of protein solution with 100 nL precipitant (1 M diammonium phosphate, 0.1 M trisodium citrate, 0.2 M sodium chloride, pH 5.5) at 20 °C. For cryoprotection during crystal harvesting, the drop was overlayed with paraffin oil, and the crystals were pulled through the oil before flash-cooling in liquid nitrogen. X-ray diffraction data collection was performed at 100 K using the ID23-1 beamline at the European Synchrotron Radiation Facility (ESRF, Grenoble, France). Data collection and refinement statistics are listed in Table 1.

### 4.3. Structure Determination, Refinement, and Analysis

The obtained diffraction data were processed using autoPROC in default settings, with a high-resolution cutoff based on *CC_1/2_* 0.60 [38]. The data were initially phased by molecular replacement using the structure of PAI-1-W175F in active conformation (PDB ID 3Q02, chain A) [39] in Phaser [40]. Next, Nb93 was modeled based on a homologous Nb structure that was selected using BLAST (PDB ID 5TP3, chain A) [41]. The structure of the PAI-1/Nb93 complex was improved by iterative rounds of manual rebuilding in Coot [42] and refinement in phenix.refine [43]. The final model has been deposited to the PDB under the accession code 6ZRV. PyMOL (The PyMOL Molecular Graphics System, version 2.0.7, Schrödinger, LLC) was used to visualize and superimpose models and to compute root-mean-squared deviations for all Cα atoms (Cα RMSD). The interface present in the complexes was analyzed using the PISA software [44].

### 4.4. Size Exclusion Chromatography with Inline Small-Angle X-Ray Scattering

SAXS experiments were performed at 288 K on the PAI-1/Nb93 complex in buffer containing 30 mM BIS-TRIS pH 5.5, 300 mM sodium chloride, and 5% *v*/*v* glycerol at the SWING beamline (SOLEIL synchrotron, Saint-Aubin, France). Protein samples (50 µL, 13 mg/mL) were loaded on a Bio SEC-3, 300 Å, 4.6 × 300 mm HPLC column (Agilent, Santa Clara, California, United States) and were eluted into the SAXS flowthrough capillary cell at a flow rate of 0.3 mL/min. SAXS data for both buffer (180 frames, 990 ms exposure time, and 10 ms dead time) and PAI-1/Nb complex (480 frames, same exposure and dead times) were collected. Buffer frames were processed, averaged, and subtracted from each protein frame (excluding outliers) using ScÅtter version 3.1r (https://bl1231.als.lbl.gov/scatter/). Scattering curves collected around the peak maximum were averaged and scaled using ScÅtter. The solution scattering profile from the atomic structure of the complex was calculated and fitted to the experimental averaged and scaled scattering curve using CRYSOL [45]. The molecular mass was estimated based on the SAXS data by dividing the Porod volume by 1.66 [46] and compared to the theoretical molecular mass based on the amino acid sequence of the PAI-1/Nb93 complex as calculated by ProtParam on the ExPASy server [47]. SAXS data has been deposited to the Small Angle Scattering Biological Data Bank under the accession code SASDJW5.

### 4.5. Activity Profile of Nb93

The functional properties of Nb93 were determined by assessing its ability to inhibit active PAI-1. PAI-1 activity was determined by a chromogenic plasminogen-coupled assay [48] and performed as previously described [23] with minor modifications. Briefly, 50 µL PAI-1-wt (84 ng/mL with an activity of ~60% of the theoretical maximum value) was incubated with an equal volume of Tris-buffered saline (TBS buffer: 50 mM TRIS base, 100 mM sodium chloride, 0.01% *v*/*v* Tween 80, pH 7.5) or with a serial two-fold dilution of Nb93, resulting in a ratio between a 0.5- and 256-fold molar excess of Nb over PAI-1 (corresponding to a Nb concentration ranging from 1 to 512 nM). Following incubation for 15 min at 37 °C, 50 µL of the mixture was transferred into the wells of a 96-well microtiter plate. Subsequently, an equal volume of tPA (20 IU/mL, a kind gift from Boehringer Ingelheim) was added and the plate was incubated at 37 °C for 15 min. After incubation, 100 µL of a substrate solution containing plasminogen (2 µM, Merck), CNBr-digested fibrinogen (1 µM, a kind gift from Roger Lijnen) and S-2403 (0.6 mM, Chromogenix, Bedford, Massachusetts, United States) were added and further incubated at 37 °C. Residual tPA activity was determined by measuring the absorbance at 405 nm. A standard curve spanning 20 U/mL to 2.5 U/mL tPA was used to quantify the amount of residual tPA activity. One hundred percent PAI-1 activity was defined as the PAI-1 activity observed in the absence of Nb93. The percentage of PAI-1 inhibition (i.e., neutralization of PAI-1 activity) was calculated from the residual PAI-1 activity measured in the presence of Nb.

### 4.6. Functional Half-Life of PAI-1

To determine the half-life of PAI-1 in the absence or presence of Nb93, PAI-1 (84 ng/mL with an activity of ~60% of the theoretical maximum value) was incubated either with TBS buffer or a 16 or 32-fold molar excess of Nb93, corresponding to 32 or 64 nM of Nb93. Nb42 was used as a negative control Nb at a 32-fold molar excess over PAI-1 (64 nM of Nb42). Aliquots of these mixtures were incubated in a water bath at 37 °C. At various time intervals, one tube of each mixture was collected and stored at −80 °C until analysis with the chromogenic plasminogen-coupled assay. To analyze the samples, the aliquots were gently thawed on ice. After transferring 50 µL of each sample to the wells of a 96-well microtiter plate, an equal volume of 20 U/mL tPA was added. The subsequent incubation and substrate reaction steps were the same as described above. One hundred percent PAI-1 activity was defined as the PAI-1 activity observed at t_0_. The percentage of PAI-1 activity at all other time points was calculated relative to the activity at t_0_. The functional half-life of active PAI-1 in the absence or presence of Nbs was calculated from the fit of the decay curve.

### 4.7. Quantification and Statistical Analysis

Quantitative data represent the mean ± standard deviation (mean ± SD, *n* ≥ 3). Inhibition curves were fitted using the non-linear regression [Inhibitor] vs. response—variable slope (four parameters) model on GraphPad Prism 7.03 (GraphPad Software, La Jolla, California, United States). Half-life curves were fitted using the non-linear regression “One phase decay” model, with y_0_ constrained to 100% remaining PAI-1 activity. Maximum inhibition, half-maximal inhibitory concentration (IC_50_), and half-life values were calculated for each experimental repeat and reported as mean ± SD (*n* ≥ 3). Statistical analyses were performed with the two-tailed unpaired *t*-test. *p*-values < 0.05 were considered statistically significant.

## Figures and Tables

**Table 1:** Data collection and refinement statistics.

PAI-1-W175F/Nb93			
Data Collection		Refinement	
Space group	*P* 4_1_ 2_1_ 2	No. of complexes/ASU	1
Cell dimensions		Reflections used in refinement	55,639 (5474)
a, b, c (Å)	117.81, 117.81, 96.48	Reflections used for *R_free_*	2315 (234)
α, β, γ (°)	90, 90, 90	*R_work_*	0.197
Resolution (Å)	52.69–1.88 (1.95–1.88)	*R_free_*	0.233
*R_merge_*	0.115 (1.326)	No. of non-hydrogen atoms	4098
*I/σ* *(I)*	12.6 (2.0)	Protein	3783
Wilson *B*-factor (Å^2^)	30.88	Water	315
*CC*_1/2_	0.998 (0.839)	Average *B*-factors (Å^2^)	
Completeness (%)	100 (100)	Protein	36.26
Redundancy	13.0 (13.1)	Water	41.16
		R.m.s. deviations	
		Bond lengths (Å)	0.007
		Bond angles (°)	0.79

Diffraction data were collected from a single crystal. The values in parentheses are for the highest resolution shell.

**Table 2:** Interactions between PAI-1 and Nb93 in the crystal structure.

PAI-1-W175F/Nb93			
Monomer	Nb93	PAI-1	Distance (Å)
Hydrogen bonds	Gln39 [HE22]*	Ser337 [OG]	2.2
	Glu44 [OE1]*	Asp181 [H]	2.1
	Glu44 [OE2]*	Ser182 [H]	2.0
	Glu44 [OE2]*	Ser182 [HG]	2.0
	Arg45 [O]*	Ile342 [H]	2.0
	Arg45 [HE]*	Ser337 [O]	2.8
	Gly47 [H]*	Ile342 [O]	1.9
	Asp62 [OD1]	Ser344 [HG]	2.1
	Trp104 [HE1]	Glu350 [OE1 ]	2.0
	Thr106 [O]	Thr205 [HG1]	2.2
	Asn108 [HD22]	Thr205 [O]	2.0
	Asn108 [HD21]	Val341 [O]	2.2
	Asn108 [OD1]	Val341 [H]	1.9
Salt bridges	Asp62 [OD2]	Arg346 [NH1]	2.8
	Glu65 [OE2]	Arg346 [NE]	3.9
	Asp99 [OD1]	Lys207 [NZ]	2.8
	Asp99 [OD2]	Lys207 [NZ]	2.7
Hydrophobic contacts ^a^	Phe37, Gly47, Cys50, Tyr59, Thr61, Trp104, Cys105, Phe107	Thr205, Pro270, Val341, Ile342, Val343, Met347, Ala348, Pro349	

Data were obtained by analyzing the interfaces present in the PAI-1-W175F/Nb42/Nb64 complex using PISA software. Residues in CDR3 are highlighted in blue. Residues in Nb FR2 are indicated by an asterisk (*), other residues belong to FR3. Residues in the RCL of PAI-1 are highlighted in red. ^a^ Only residues highly involved in hydrophobic interactions at the interaction interface.
